# Risk stratification system and visualized dynamic nomogram constructed for predicting diagnosis and prognosis in rare male breast cancer patients with bone metastases

**DOI:** 10.3389/fendo.2022.1013338

**Published:** 2022-11-11

**Authors:** Bing Gao, Xiao-lan Ou, Mu-feng Li, Meng-die Wang, Fei Huang

**Affiliations:** ^1^ Department of Orthopedics, China-Japan Union Hospital of Jilin University, Changchun, China; ^2^ Department of Hand Surgery, The Second Hospital of Jilin University, Changchun, China; ^3^ Department of Orthopedics, The Second Hospital of Jilin University, Changchun, China; ^4^ Department of Neurology, China-Japan Union Hospital of Jilin University, Changchun, China

**Keywords:** bone homeostasis, male, breast cancer, bone metastasis, overall survival, cancer-specific survival, risk stratification, dynamic nomogram

## Abstract

**Background:**

Bone metastases (BM) from malignant tumors could disrupt the balance between osteoclasts and osteoblasts and affect bone homeostasis. Malignant breast cancer (BC) is rare in male patients, and co-occurrence of BM is even rarer. Given its low incidence, there is limited research evaluating risk and prognosis. Despite the widespread application of nomograms to predict uncommon malignancies, no studies have constructed predictive models focusing on the diagnosis and prognosis of male breast cancer with bone metastases (MBCBM).

**Methods:**

This study selected all male breast cancer patients (MBC) between 2010 and 2019 in the Surveillance, Epidemiology, and End Results (SEER) database. We used simple and multivariate Logistic regression analyses to identify independent risk factors for BM in MBC patients. Then simple and multivariate Cox regression analyses were employed to determine the independent prognostic factors for overall survival (OS) and cancer-specific survival (CSS) in MBCBM patients. We established and validated three new nomograms based on these independent factors.

**Result:**

A total of 4187 MBC patients were included, with 191 (4.56%) having bone metastases at the time of diagnosis. The independent risk factors of BM in MBC patients included age, tumor size, marital status, T stage, and N stage. In MBCBM patients, independent prognostic factors for OS and CSS were both age, T stage, ER status, PR status, and surgery. The concordance index (C-index), the area under the curve (AUC) of the receiver operating characteristic curve (ROC), the calibration curve, and the decision curve analysis (DCA) confirmed that these three nomograms could accurately predict the diagnosis and prognosis of MBCBM patients with excellent discrimination and clinical utility superior to the TNM staging system. We then established two prognostic-based risk stratification systems and three visualized dynamic nomograms that could be applied in clinical practice.

**Conclusion:**

In conclusion, this study aimed to establish and validate an accurate novel nomogram to objectively predict the diagnosis and prognosis of MBCBM patients. On this basis, prognostic-based risk stratification systems and visualized dynamic nomograms were constructed to facilitate doctors and patients to quantify individual BM risk probability and survival probability to assist in personalized risk assessment and clinical decision-making.

## Introduction

Breast cancer (BC) is the most common cancer in women, accounting for 30.79% of the female cancer population, according to the latest annual cancer statistics report by the American Cancer Society (1). Nevertheless, male breast cancer (MBC) is sporadic compared to female breast cancer(FBC), with an incidence of only 0.5% to 1% during the last 40 years and minimal improvement in survival (2). In 2022, there will be 2710 new cases of male breast cancer diagnosed in the United States, accounting for 0.28% of the male cancer population, with incidence and mortality rates of 0.93% and 1.21% among all BC patients, respectively (1). MBC patients exhibit biological differences from FBC patients and are generally associated with older age, higher grade, later stage, more distant metastases, and more ominous prognosis (3, 4).

In BC patients, distant metastases have become the leading cause of death (90%), with bone metastases (BM) being the most prevalent (65-80%) (5, 6). BC with BM frequently causes skeletal-related events. This is because breast cancer cells enter the bone marrow through interactions with endothelial cells and osteoblasts, disrupting the balance between osteoclasts and osteoblasts, affecting bone homeostasis, and subsequently leading to skeletal dysfunction, such as spinal cord compression, pathological fracture osteosclerosis, osteoporosis, osteoarthritis (7, 8). As a result, patients’ median survival time was reduced to 24-55 months following BM diagnosis (9, 10). This has a substantial detrimental impact on patients’ quality of life and mental health, as well as increasing social burden and triggering public anxiety. To date, there are many published reports on FBC with BM, but few on the clinical characteristics and prognostic survival of MBC with BM (MBCBM) (11–14). Given the extremely low incidence, most previous studies on MBCBM were case reports with too small sample sizes, short follow-up time, and insufficient reliability. Fortunately, the Surveillance, Epidemiology, and End Results (SEER) database, which covers approximately 30% of the U.S. population, could facilitate researchers comprehensively analyzing tumor characteristics, diagnostic staging, and survival status of MBCBM patients based on extensive data samples (15).

The key to in-depth research on malignancies has consistently been predicting prognosis. The traditional TNM staging system (American Joint Committee on Cancer, AJCC) is the most widely used tool for predicting the prognosis of malignancies (16, 17). However, the TNM staging system excludes critical clinical factors, including age, histological subtype, and treatment information. It is unsuitable for quantifying the risk probability of BM in patients with MBC and the likelihood of survival in patients with MBCBM (18, 19). The nomogram has been extensively applied in diagnosing and prognostic research of malignant tumors. By integrating significant clinical variables and tumor characteristics, it could intuitively display risk factors and survival probability in a simple graph, assist individualized risk assessments and clinical decision-making, and make up for the deficiencies of the TNM staging system (17).

Based on the SEER database, this study was the first to establish and validate practical nomograms for predicting the diagnosis and prognosis of BM in MBC patients. These nomograms demonstrated outstanding predictive accuracy and clinical utility, allowing doctors and MBC patients to quickly identify risk factors for BM, quantify individual BM risk probability and survival probability, then determine tailored treatment and follow-up strategies.

## Materials and methods

### Data source and variable definitions

Each year, the Surveillance, Epidemiology, and End Results (SEER) database collects information on 450,000 patients with rare malignancies for free studies by the global academic community to advance cancer breakthrough research and public health education (20). After the application was approved, we selected a total of 4187 MBC patients required for this study from the database, including 191 MBCBM patients. Notably, we found that the most recent SEER plus database submitted in November 2021 only had patient data from seventeen registries, so we replaced the missing Detroit data with the data presented in November 2020, and ensured that there were no duplicates of patient IDs. Inclusion criteria were as follows (1): confirmed diagnosis by positive histological evidence (2); the malignant tumor category was breast cancer (3) diagnosed from 2010 to 2019 (4); the gender was male. Exclusion criteria were as follows (1): Demographic variables (age, sex, race, marital status) were unknown (2) Tumor characteristics (breast cancer subtype, ER status, PR status, tumor size, laterality, grade, T stage, N stage) were unknown.

This study collected the following demographic and clinical information: age, race, marital status, year of diagnosis, primary site, histological subtype, breast cancer subtype, ER Status, PR Status, laterality, tumor size, grade, T stage, N stage, brain metastases, lung metastases, liver metastases, surgery, radiotherapy, and chemotherapy. Grouping continuous variables (tumor size and age) facilitated further analysis. We refer to the provisions of the American Joint Committee on Cancer Eighth Edition Cancer Staging Manual for the pathological staging of primary breast cancer, and divide the tumor size into three groups (<20, 20-50, >50) (21). We obtained age groups (20-39, 40-59, 60-79, ≥80) regarding the age brackets set by the American Cancer Society for the convenience of statistical analysis of cancer patients (1). In marital status, “Alone” included divided, separated, single, unmarried, or widowed. Due to the small sample size of several histological subtypes, we combined and categorized them by the International Classification of Diseases for Oncology, 3rd Edition (ICD-O-3) codes. “Others” included histological subtypes other than Infiltrating duct carcinoma. Among the breast cancer subtypes, “Luminal A” denoted HR+/HER2-, “Luminal B” denoted HR+/HER2+, and “Others” denoted HR-/HER2-, HR-/HER2+. The “Peripheral portion” in the primary site of the breast contained the upper-inner, lower-inner, upper-outer, and lower-outer quadrant and axillary tail. “Breast, NOS” represented the breast of an unspecified location cancer. We divided tumor grade into low-grade (I, II) and high-grade (III/IV). “Grade I” indicated well-differentiated, “grade II” marked moderate differentiation, and “grade III” denoted poor differentiation, “Grade IV” meant undifferentiated. There are very few patients with MBCBM in grade IV, so we combined it with grade III. According to the recent SEER Program Coding and Staging Manual (https://seer.cancer.gov/manuals/2023/appendixc.html), we categorized surgical options for MBC patients into no surgery, breast-conserving surgery, partial mastectomy, and radical mastectomy. “Breast-conserving surgery” included surgery codes: 20-24, “Partial mastectomy” comprised: 30,40–49, and “Radical mastectomy” represented 50–59,60-72”. We identified overall survival (OS) and cancer-specific survival (CSS) as the primary endpoints. OS denoted the time between the initial diagnosis and the last follow-up (including death regardless of cause). CSS was defined as the same period, but only if death is due to MBCBM.

### Statistical analysis

All statistical analyses were performed in this study by R software (version 4.1.2), with a P-value of <0.05 (two-sided) defined as statistical test significance. For risk factors for BM in MBC patients, we utilized simple logistic regression analysis to screen for all variables with P<0.05. These significant variables were then incorporated into the multivariate logistic analysis to identify independent risk factors for developing BM in MBC patients. We constructed and validated a diagnostic nomogram for BM in MBC patients based on the above risk factors. For prognosis in MBCBM patients, we applied simple and multivariate Cox regression analysis to identify all independent prognostic factors for OS and CSS. Based on the above prognostic factors, we constructed and validated two new nomograms for predicting 12-, 24-, and 36-month OS and CSS in MBCBM patients, respectively. The accuracy and discrimination of the above three nomograms were verified by the concordance index (C-index), the area under the curve (AUC) in the receiver operating characteristic (ROC) curve, and the calibration curve. Then we drew a decision curve analysis (DCA) to assess the net clinical benefit of nomograms (22).

X-tile software (version 3.6.1, Yale University School of Medicine, USA) could visualize the optimal cutoff point for continuous variables based on the highest χ2 value defined by Kaplan-Meier survival analysis and log-rank test to reveal the association between expression tumor markers and patients’ prognosis, which is often applied to age, tumor size, the number of lymph nodes, risk scores, etc. (23, 24). For each MBCBM patient, we calculated two prognostic nomogram scores (i.e., risk scores) separately, using X-tile analysis to quickly determine the optimal cutoff for risk scores (23). Based on these cutoffs of OS and CSS, we grouped MBCBM patients into three categories to establish two risk stratification systems. Kaplan-Meier curves and log-rank tests verified actual differences in OS and CSS for each risk group. Furthermore, we applied the “DynNom” R package to develop three visualized dynamic nomograms that facilitate doctors and MBC patients to assess risk probabilities quickly and accurately for BM, as well as OS and CSS for MBCBM patients.

## Results

### Patient characteristics

**Figure 1:**
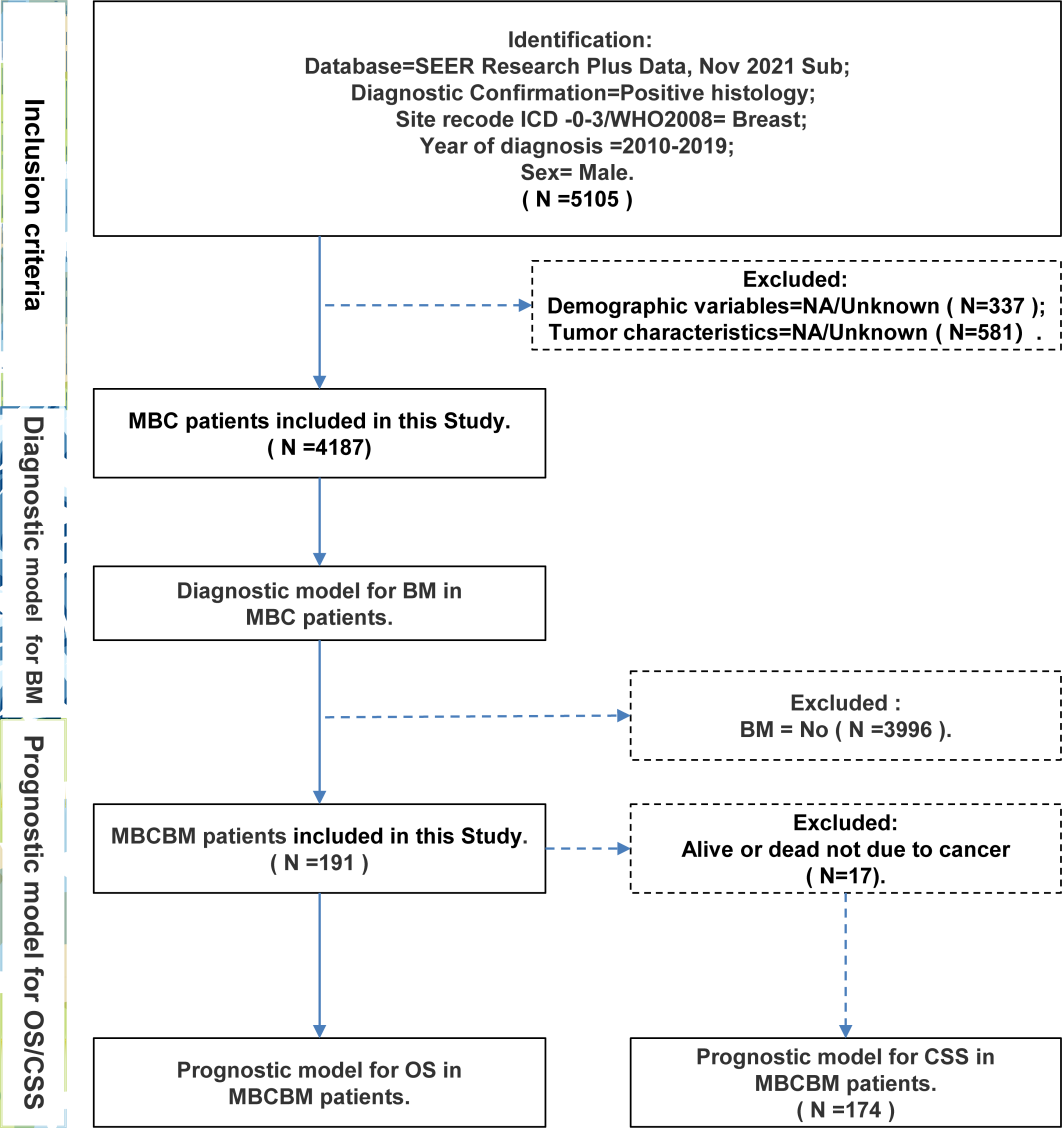
The flow chart for patient selection and study design (MBC, male breast cancer; BM, bone metastases; MBCBM, Male breast cancer with bone metastases; OS, overall survival; CSS, cancer-specific survival).

This study selected MBC patients between 2010 and 2019 from the SEER database. **Figure 1** depicted a flowchart of the patient selection procedure. Among 4187 MBC patients, 191 developed BM. Based on all MBC patients, we constructed a diagnostic model of BM. Based on all MBCBM patients, we established prognostic models for OS and CSS. **Table 1** summarized the demographic and clinical characteristics of all selected patients. All MBCBM patients were older than 20 years, with a median age of 68 years, mainly between 60 and 79 years (104, 54.5%). Tumor sizes were mostly between 20 and 50 mm (108, 56.5%) (**Table 1**). Among the identified cases, MBCBM patients were predominantly white (139,79.8%) and married (104,54.5%), with a slight increase in cases between 2015 and 2019 compared to 2010-2014. The most common primary site in MBCBM patients was the central portion (82, 42.9%), while overlapping lesions (28, 14.7%) were relatively infrequent. The risk of developing BC was nearly comparable on the left (98,51.3%) and right (93,48.7%). Most histological subtypes in MBCBM patients (174, 91.1%) were infiltrating duct carcinoma. The most common breast cancer subtype was Luminal A (136, 71.2%), while Luminal B was uncommon (33, 17.3%). Most estrogen receptors (ER) and progesterone receptors (PR) were positive among MBCBM patients, accounting for 92.7% and 82.7%, respectively. In terms of tumor grade, low-grade(I/II) MBC accounted for most non-BM MBC patients (2615, 65.4% in total). Among MBCBM patients, the proportion of high-grade(III/IV) patients increased (103, 53.9%), virtually matching that of low-grade patients (88, 46.1%).

**Table 1:** Baseline demographic and clinicopathological characteristics of all MBC patients.

Characteristics	Non-bone metastases N=3749, N (%)	Bone metastases N=236, N (%)	All N=3985, N (%)
**Age(years)**			
Mean (SD)	67.6 (12.2)	63.3 (13.3)	67.4 (12.3)
Median [Min, Max]	68.0 [22.0, 99.0]	64.0 [31.0, 88.0]	68.0 [22.0, 99.0]
**Age(years)**			
≥80	727 (18.2%)	21 (11.0%)	748 (17.9%)
20-39	62 (1.6%)	10 (5.2%)	72 (1.7%)
40-59	928 (23.2%)	56 (29.3%)	984 (23.5%)
60-79	2279 (57.0%)	104 (54.5%)	2383 (56.9%)
**Race**			
Black	572 (14.3%)	38 (19.9%)	610 (14.6%)
Others	237 (5.9%)	14 (7.3%)	251 (6.0%)
White	3187 (79.8%)	139 (72.8%)	3326 (79.4%)
**Marital status**			
Alone	1200 (30.0%)	87 (45.5%)	1287 (30.7%)
Married	2796 (70.0%)	104 (54.5%)	2900 (69.3%)
**Year of diagnosis**			
2010-2014	1864 (46.6%)	85 (44.5%)	1949 (46.5%)
2015-2019	2132 (53.4%)	106 (55.5%)	2238 (53.5%)
**Primary site**			
Breast, NOS	462 (11.6%)	43 (22.5%)	505 (12.1%)
Central portion	1999 (50.0%)	82 (42.9%)	2081 (49.7%)
Overlapping lesion	652 (16.3%)	28 (14.7%)	680 (16.2%)
Peripheral portion	883 (22.1%)	38 (19.9%)	921 (22.0%)
**Histological subtype**			
Infiltrating duct carcinoma	3418 (85.5%)	174 (91.1%)	3592 (85.8%)
Others	578 (14.5%)	17 (8.9%)	595 (14.2%)
**Breast cancer subtype**			
Luminal A	3312 (82.9%)	136 (71.2%)	3448 (82.4%)
Luminal B	441 (11.0%)	33 (17.3%)	474 (11.3%)
Others	243 (6.1%)	22 (11.5%)	265 (6.3%)
**ER status**			
Negative	89 (2.2%)	14 (7.3%)	103 (2.5%)
Positive	3907 (97.8%)	177 (92.7%)	4084 (97.5%)
**PR status**			
Negative	344 (8.6%)	33 (17.3%)	377 (9.0%)
Positive	3652 (91.4%)	158 (82.7%)	3810 (91.0%)
**Laterality**			
Left	2121 (53.1%)	98 (51.3%)	2219 (53.0%)
Right	1875 (46.9%)	93 (48.7%)	1968 (47.0%)
**Tumor size(mm)**			
<20	1635 (40.9%)	18 (9.4%)	1653 (39.5%)
>50	202 (5.1%)	65 (34.0%)	267 (6.4%)
20-50	2159 (54.0%)	108 (56.5%)	2267 (54.1%)
**Grade**			
Grade I	489 (12.2%)	9 (4.7%)	498 (11.9%)
Grade II	2126 (53.2%)	94 (49.2%)	2220 (53.0%)
Grade III/IV	1381 (34.6%)	88 (46.1%)	1469 (35.1%)
**T stage**			
T1	1840 (46.0%)	22 (11.5%)	1862 (44.5%)
T2	1749 (43.8%)	74 (38.7%)	1823 (43.5%)
T3	108 (2.7%)	27 (14.1%)	135 (3.2%)
T4	299 (7.5%)	68 (35.6%)	367 (8.8%)
**N stage**			
N0	2318 (58.0%)	42 (22.0%)	2360 (56.4%)
N1	1189 (29.8%)	94 (49.2%)	1283 (30.6%)
N2	325 (8.1%)	32 (16.8%)	357 (8.5%)
N3	164 (4.1%)	23 (12.0%)	187 (4.5%)
**Brain metastasis**			
No	3992 (99.9%)	182 (95.3%)	4174 (99.7%)
Yes	4 (0.1%)	9 (4.7%)	13 (0.3%)
**Lung metastasis**			
No	3950 (98.8%)	128 (67.0%)	4078 (97.4%)
Yes	46 (1.2%)	63 (33.0%)	109 (2.6%)
**Liver metastasis**			
No	3984 (99.7%)	172 (90.1%)	4156 (99.3%)
Yes	12 (0.3%)	19 (9.9%)	31 (0.7%)
**Surgery**			
No	215 (5.4%)	115 (60.2%)	330 (7.9%)
Breast-conserving surgery	490 (12.3%)	10 (5.2%)	500 (11.9%)
Partial mastectomy	2050 (51.3%)	29 (15.2%)	2079 (49.7%)
Radical mastectomy	1241 (31.1%)	37 (19.4%)	1278 (30.5%)
**Radiation**			
No	2857 (71.5%)	113 (59.2%)	2970 (70.9%)
Yes	1139 (28.5%)	78 (40.8%)	1217 (29.1%)
**Chemotherapy**			
No	2552 (63.9%)	88 (46.1%)	2640 (63.1%)
Yes	1444 (36.1%)	103 (53.9%)	1547 (36.9%)

T2 (38.7%) and T4 (35.6%) were the most common T stages, while T1 (11.5%) and T3 (14.1%) were relatively uncommon. As for the N stage, most MBCBM patients (49.2%) were in N1, 22.0% in N0, 16.8% in N2, and 12.0% in N3. Brain metastases (0.1%), lung metastases (1.2%), and liver metastases (0.3%) were rare in non-BM MBC patients. However, brain metastases (4.7%), lung metastases (33.0%), and liver metastases (9.9%) were relatively more prevalent in MBCBM patients. Regarding treatment, among non-BM MBC patients, 94.6%, 28.5%, and 36.1% underwent surgery, radiotherapy, and chemotherapy, respectively. The probability of receiving partial mastectomy and radical mastectomy, and breast-conserving surgery were 51.3%, 31.1%, and 12.3%, respectively. MBCBM patients, in contrast, appeared to have a more negative willingness to undergo surgery, at only 39.8%, compared to 40.8% and 53.9% for radiotherapy or chemotherapy, respectively. The probability of receiving partial mastectomy, radical mastectomy, and breast-conserving surgery were 19.4%, 15.2%, and 5.2%, respectively.

### Development and validation of the nomogram for BM

**Figure 2:**
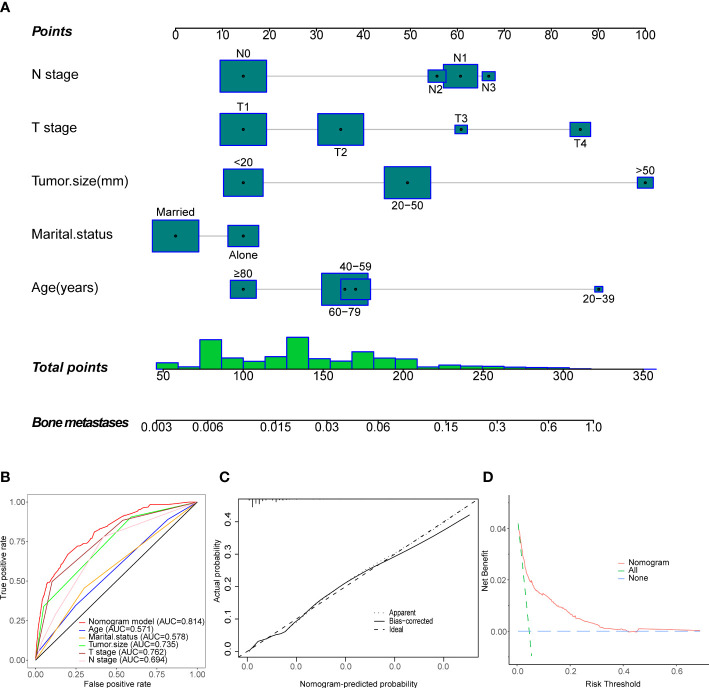
Establishment and validation of a diagnostic nomogram to estimate the risk of BM in MBC patients. **(A) **The diagnostic nomogram. **(B)** The receiver operating characteristic curve (ROC). **(C) **The Calibration curve. **(D) **The decision curve analysis (DCA).

At the time of initial diagnosis, 191 (4.56%) of all MBC patients were confirmed as BM, while 3749 (95.44%) as non-BM. This study utilized simple and multivariate Logistic regression to analyze fourteen potential factors. The results revealed five independent risk factors for BM, including age, tumor size, marital status, T stage, and N stage, as shown in **Supplementary Table S1**. It is worth noting that newly diagnosed MBC patients might not even have access to therapeutic interventions (including surgery, radiotherapy, or chemotherapy). It seems difficult to obtain information on brain, lung, and liver metastases simultaneously until bone metastases are identified. Therefore, we did not incorporate them in the formation of the diagnostic model to avoid biased results. Based on the above independent risk factors, we developed the first new nomogram to predict the probability of developing BM in MBC patients (**Figure 2A**).

To estimate the patient’s likelihood of BM, readers could generate a total score based on the scores for each MBC patient’s independent risk factors and draw a vertical line between the “Total Score” and “Bone Metastasis” axes. Then we calculated the C-index and the AUC of the ROC, both were 0.814 (0.784, 0.844) (**Figure 2B** **),** demonstrating that the nomogram has solid predictive performance compared to each independent variable. The calibration curve revealed excellent agreement between the actual survival probability and the nomogram’s prediction (**Figure 2C**). According to the DCA curve, the nomogram exhibited a high net benefit and clinical value (**Figure 2D**).

### Development and validation of nomograms for OS and CSS

**Figure 3:**
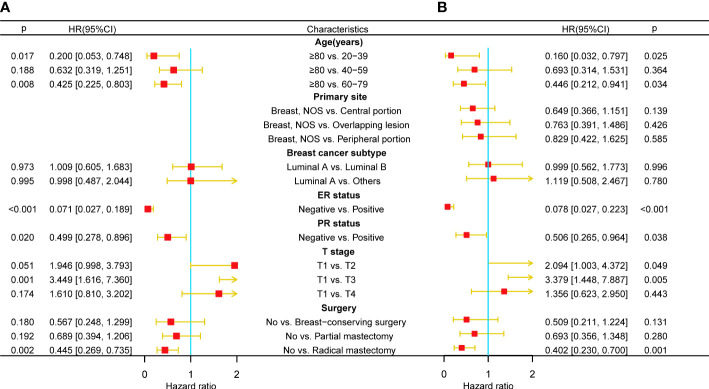
Multivariate COX regression forest plots for OS and CSS in MBCBM patients. **(A) **The forest plot for OS. **(B) **The forest plot for CSS.

**Figure 4:**
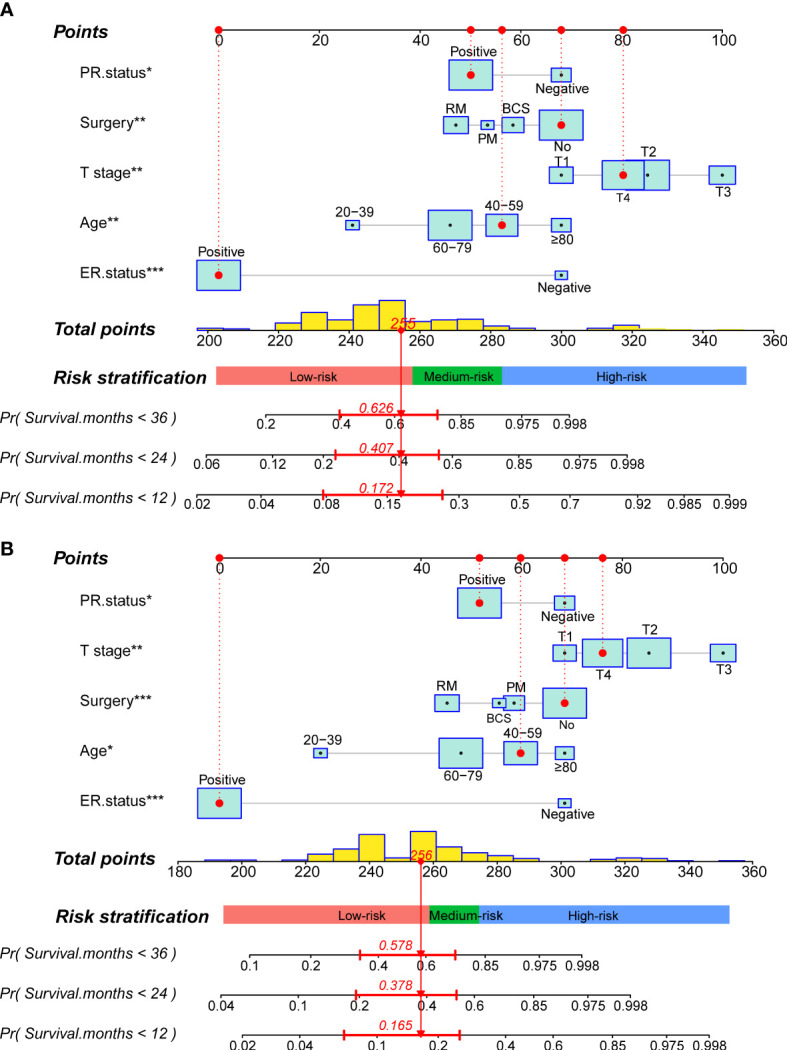
Construction of two prognostic nomograms for predicting the 12-,24- and 36months OS and CSS in MBCBM patients. **(A) **The prognostic nomogram for OS. **(B) **The prognostic nomogram for CSS (***:P < 0.001; **:P < 0.01; *:P < 0.05; BCS, breast-conserving surgery; PM, partial mastectomy; RM, radical mastectomy).

Only 191 qualified MBCBM patients were included in the prognostic research because of the infrequent incidence (**Figure 1**). We employed simple and multivariate Cox regression methods on twenty potentially correlated variables to identify independent prognostic variables and then drew forest plots for OS and CSS respectively (**Supplementary Tables 2**, **3** and **Figures 3A, B**). Age, T stage, ER status, PR status, and surgery were five independent prognostic variables for OS (**Figure 3A**). In the simple COX regression analysis of CSS, the P-values for both subgroups of age (≥80 vs. 20-39) and primary site (Breast, NOS vs. Central portion) were both precisely equal to 0.05. To avoid missing independent variables, we also included them in a multiple COX regression analysis. Age, T stage, ER status, PR status, and surgery were identified as independent prognostic variables for CSS (**Figure 3B**). We developed the following nomograms to predict 12-, 24-, and 36-month OS and CSS in MBCBM patients based on the above independent prognostic variables, respectively (**Figures 4A, B**).

Readers could calculate survival probability by drawing vertical lines between the “Total Score” axis and the axis of survival probability at 12, 36, and 60 months based on the scores for each MBCBM patient’s independent prognostic variables. As depicted in **Figures 4A, B**, the given patient was a 49-year-old T4-stage MBC case with ER-positive, PR-positive, and no surgery. The total nomogram score for OS was 255 points, with survival probabilities of 82.8%, 59.3%, and 37.4% at 12, 36, and 60 months, respectively. The total nomogram score for CSS was 256 points, with survival probabilities of 83.5%, 62.2%, and 42.2% at 12, 36, and 60 months, respectively.

**Figure 5:**
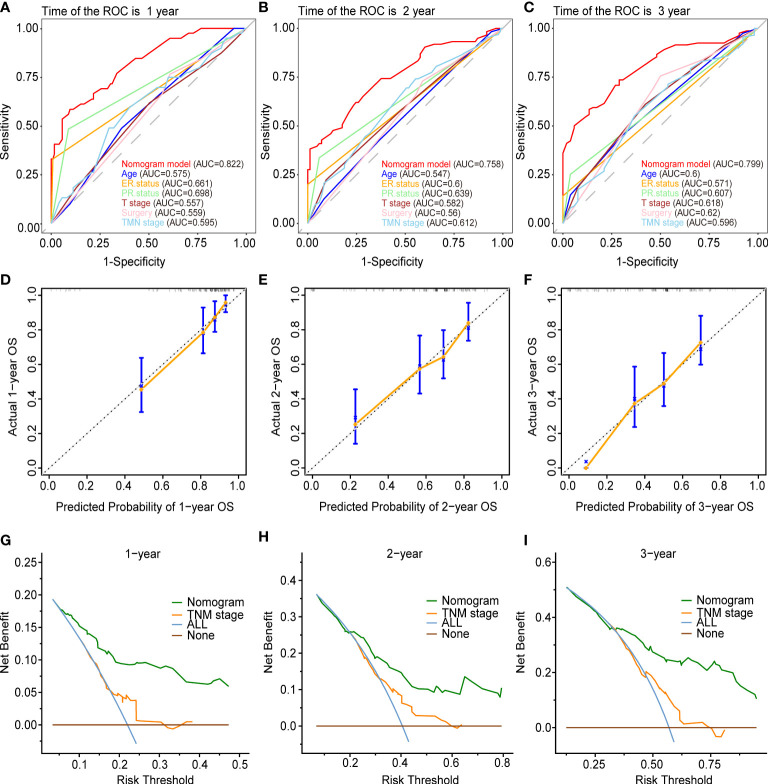
Validation of the prognostic nomogram for the 12-,24- and 36months OS. **(A–C) **The receiver operating characteristic curves (ROC). **(D–F)** The calibration curves. **(G–I)** The decision curve analyses (DCA).

**Figure 6:**
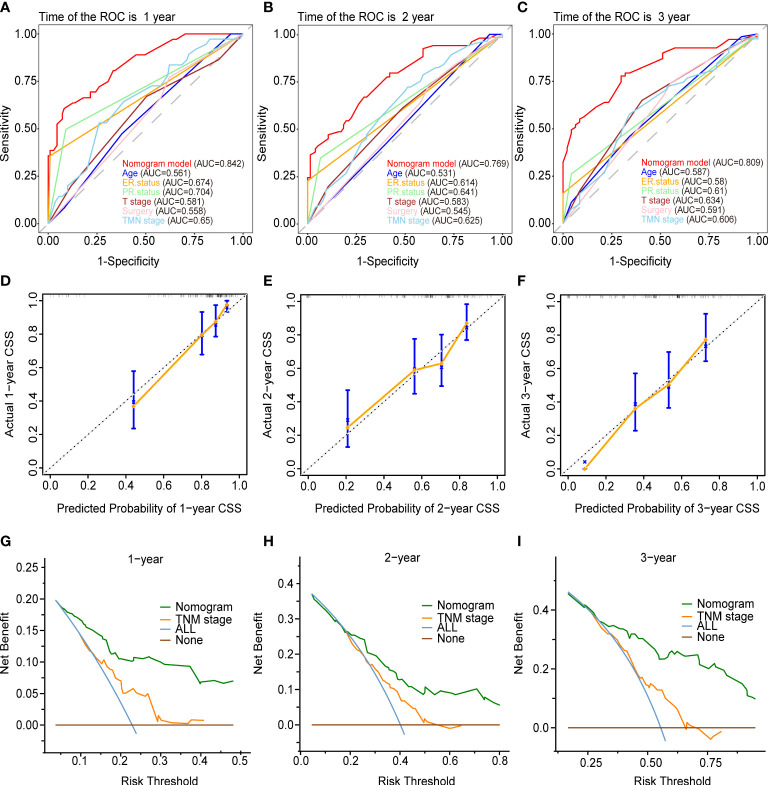
Validation of the prognostic nomogram for the 12-,24- and 36months CSS. **(A–C)** The receiver operating characteristic curves (ROC). **(D–F)** The calibration curves. **(G–I)** The decision curve analyses (DCA).

**Figures 5** and **6** demonstrated what we plotted ROC, calibration curve, and DCA to validate the predictive ability of the two prognostic nomograms. The nomogram for OS had a significantly higher C-index than the TMN staging system ((HR: 0.72, 95%CI: 0.694-0.746) vs. (HR: 0.583, 95%CI: 0.552-0.614)). Furthermore, the AUCs of the ROC at 12-, 24-, and 36 months were much higher than those of the TMN staging system (0.822, 0.758, 0.799 vs. 0.595, 0.612, 0.596). Both the C-index and the AUCs exceeded 0.72, suggesting that the OS nomogram performed substantially better than the AJCC staging system in prediction (**Figures 5A–C**). The same went for the nomogram for CSS. The CSS nomogram had a significantly higher C-index than the TMN staging system ((HR: 0.74, 95%CI: 0.713-0.767) vs. (HR:0.611, 95%CI: 0.579-0.642)). Furthermore, the AUCs of the ROC at 12-, 24-, and 36 months were much higher than those of the TMN staging system (0.842, 0.769, 0.809 vs. 0.65, 0.625, 0.606). The CSS nomogram performed substantially better than the TMN staging system in prediction, as evidenced by both the C-index and the AUCs over 0.74. (**Figures 6A–C** **).** The calibration curves revealed a good agreement between the two nomograms’ predicted survival probabilities and the actual outcomes (**Figures 5D–F**, **6D–F** **).** The nomograms’ curves in DCA were substantially higher than the TMN staging system’s, indicating that both nomograms have considerable net benefits and clinical utilities over the traditional TMN staging system (**Figures 5G–I**, **6G–I**).

### Risk stratification system

**Figure 7:**
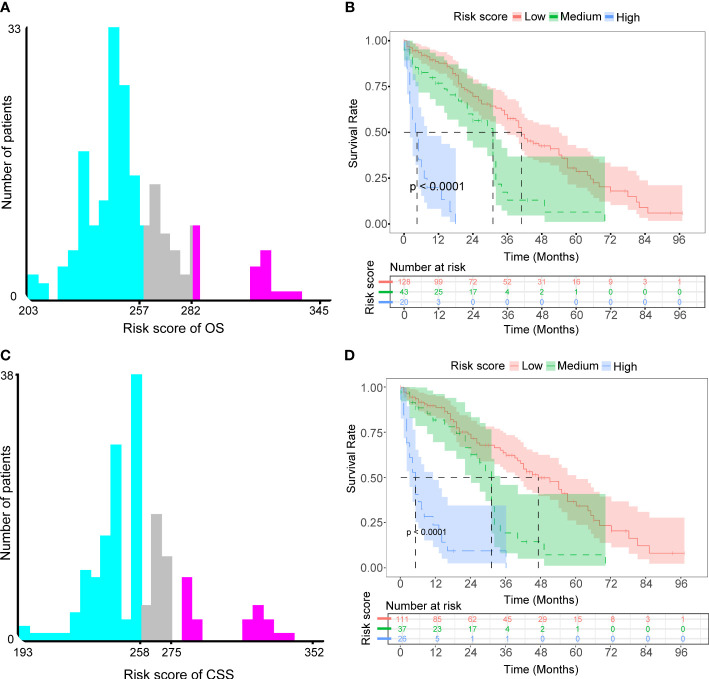
Determined optimal cutoff points for risk scores to construct two risk stratification systems for OS and CSS. **(A)** X-tile software identified the optimal cutoff point for risk scores for OS by the histogram. **(B)** Kaplan-Meier survival analysis plotted prognostic curves among distinct risk groups for OS. **(C)** X-tile software identified the optimal cutoff point for risk scores for CSS by the histogram. **(D)** Kaplan-Meier survival analysis plotted prognostic curves among distinct risk groups for CSS.

We defined each patient’s total nomogram score as the risk score and determined the optimal cutoff for risk scores for all patients by the X-tile procedure (**Figures 7A, C**). The colors of the histograms represent the association of each risk group with prognosis, from left to right representing low, intermediate, and high risk. The height of the histogram indicates the corresponding number of patients. The intersection point between the different colored bars on the horizontal axis is the best cutoff value. For OS, all patients could be divided into three groups: low-risk (N=128, 67.01%, scores<257), medium-risk (N=43, 22.51%, scores between 257 and 282) and high-risk (N=20, 10.47%, scores>282). For CSS, all patients can be divided into three groups: low-risk (N=111, 64.16%, scores <258), medium-risk (N=37, 21.39%, scores between 258 and 275) and high-risk (N=26, 15.03%, scores>275). KM survival curves and log-rank tests validated significant differences (p<0.001) between all three risk groups for OS and CSS, indicating the validity of the nomogram-based risk stratification system (**Figures 7B, D**).

### Visualized dynamic nomogram

We developed three visualized dynamic nomograms for predicting the risk probability of BM in patients with the initial diagnosis of MBC and for predicting OS and CSS in MBCBM patients. Through routine clinical factors, doctors and MBC patients could quickly and intuitively assess individual BM risk and survival probability (**Supplementary Figures S1**–**3**). For example, we hypothesized to include a 55-year-old married MBC patient with 60-mm-sized, T4N1, ER-positive, PR-positive breast cancer who underwent a radical mastectomy. As shown in **Supplementary Figure S1**, the probability of developing BM in this patient without distant metastasis was 34.7% (23.8%, 47.5%). **Supplementary Figures S1** and **S2** demonstrate that the estimated survival probabilities for OS at 12-, 24-, 36-, 48-, and 60 months for this given patient were 95%, 87%, 76%, 67%, and 54%, respectively. The estimated survival probabilities for CSS were 95%, 87%, 77%, 69%, and 56%, respectively.

(https://gaobing191.shinyapps.io/Nomogram_for_Diagnosis_of_MBCBM/)

(https://gaobing191.shinyapps.io/Nomogram_of_OS_in_MBCBM/)

(https://gaobing191.shinyapps.io/Nomogram_of_CSS_in_MBCBM/)

## Discussion

Malignant tumor cells interfere with bone microenvironment homeostasis and cause malignant tumor bone metastasis, which in turn aggravates the imbalance of bone homeostasis and generates a series of adverse skeletal events. MBC patients have a 1.75 times higher rate of distant metastasis than female patients, with BM being the most prevalent (4). The poor prognosis is frequently associated with distant metastasis. It has previously been reported that once BC patients were diagnosed with BM, they were generally at an advanced stage of malignancy, with multiple distant organ metastases (25). The median life expectancy would be drastically decreased to 2-3 years (3). Most MBC patients are unconcerned about BM and will only undergo further examination if signs or symptoms are prominent. However, MBCBM patients are less likely than women to accomplish the whole course of standard treatment at this time, resulting in poor adherence and worse overall mortality rates (26).

Therefore, if we timely assess the risk probability of BM in MBC patients and the survival probability of MBCBM patients in early screening, we could achieve early prevention and clinical intervention to prolong survival time. Zhou et al. analyzed the prognostic factors of MBCBM patients but overlooked significant clinical variables of BC such as ER, PR, and primary site, and did not construct effective diagnostic and prognostic models (14). In this study, combined with clinical practice, we incorporated more variables related to MBC. We identified independent risk factors for BM in patients with MBC and independent prognostic factors for patients with MBCBM. In previous studies on MBC patients, nomograms have been validated with better predictive performance than traditional AJCC staging systems (27, 28). Nevertheless, to the best of our knowledge, this is the first study to establish and validate a diagnostic nomogram, prognostic nomogram, and risk stratification system to accurately predict the risk probability of BM in MBC patients and OS and CSS in MBCBM patients.

In this study, MBCBM patients, like all MBC patients, shared multiple similar clinicopathological characteristics, including age, ethnicity, laterality, ER status, PR status, histological subtype, BC subtype, etc. MBCBM patients, on the other hand, had larger tumors, higher grades, later T/N stages, higher risks of metastasis, higher possibilities of non-surgical treatment, and shorter OS and CSS. In MBC patients, age and tumor size have been widely reported as independent prognostic factors (19, 27, 28). MBCBM patients with tumors more significant than 50 mm accounted for 34% of the study (**Figure 1**). The larger the tumor size, the more possible it is to develop BM. Age played an essential role in the occurrence and prognosis of BM in MBC patients. Notably, in our study, the younger the age at developing MBC, the greater the likelihood of BM. In general, the older the MBCBM patients, the higher the nomogram score, and the lower the prognostic survival rate. But interestingly, the prognosis for patients in the 60-79 age group was even better than that of the 40-59 age group. The T stage refers to tumor size or location, while the N stage refers to regional lymph node involvement in the TNM staging system (16). In MBC patients, the higher the stage of T and N, the more susceptible to developing BM. T stage also significantly affects the prognosis of MBCBM patients. The later the T stage is, the higher the nomogram score and the lower the prognosis survival rate. But in this study, patients with the T4 stage had a better prognosis than T2 and T3 stages.

BC is a hormone-sensitive tumor. Vargas et al. found that estrogen-related receptor alpha (ERRα), which guides the migration of BC cells from the primary site into the bone microenvironment, disrupts the balance between bone formation and bone resorption, which in turn affects bone homeostasis (8, 29). It has been reported that hormone receptor levels in MBC patients are significantly higher than in FBC patients (27, 28). In the present study, ER status and PR status were two independent prognostic factors in MBCBM patients. Risk scores of ER-negative or PR-negative MBCBM patients were substantially higher than those of ER-positive or PR-positive in the prognostic nomograms of OS and CSS, suggesting a poor prognosis. It is consistent with the study of Chen et al. (28). ER status and PR status may be helpful in identifying biological targets for the prevention and treatment of MBCBM.

Many studies have suggested that histological subtype, primary site, and BC subtype are independent predictors of BC patient diagnosis or prognosis (11–13). In this study, although simple analysis indicated that these variables might affect the diagnosis and prognosis of MBCBM patients, multivariate analysis revealed no significance (P>0.05). Therefore, they have not been incorporated into our nomograms. The article from Zhou et al. also supported this result (14). Metastasis in one organ might accelerate the spread of cancer cells to other organs, and BM could considerably enhance the risk of other metastases and vice versa (4, 18). In our study, the metastatic rates of MBCBM patients in the liver, lung, and brain were 4.7%, 33%, and 9.9%, respectively. Brain metastases represent a catastrophic event, with a median survival of only 3-25 months, even when patients receive whole-brain radiotherapy (WBRT) (6). However, in this study, brain, liver, and lung metastasis were not independent prognostic factors in MBCBM patients (P>0.05). This might be due to statistical bias caused by the small sample size.

In our study, the proportion of MBCBM patients who underwent surgery (39.8%) was significantly lower than that of all MBC patients (94.6%). And the proportion who received adjuvant therapy (radiotherapy, 40.8%; chemotherapy, 53.9%) was significantly higher than that of all MBC patients (radiotherapy, 28.5%; chemotherapy, 36.1%), indicating that most MBCBM patients prefer non-surgical treatment. However, after performing the multivariate COX regression analysis and presenting the final nomograms, we noticed that surgery substantially improved prognosis in MBCBM patients (HR<1, P<0.05), which was consistent with previous studies (19, 27, 28, 30). Radical mastectomy is significantly better than breast-conserving surgery and partial mastectomy and is the best surgical option. Therefore, when health conditions permit, and the surgical indications are met, we recommend that primary-site surgery for resectable breast cancer should be performed as far as possible. Timely surgery may help control the spread of tumor cells to the surrounding area to prolong survival time and improve quality of life.

Unfortunately, patients with metastatic MBC often lose the opportunity for surgery due to poor overall health and prefer palliative care (chemotherapy, radiotherapy, endocrine therapy, etc.) to increase life expectancy (30). However, many studies in recent years have shown that primary tumor surgery can also be used as palliative care to control tumor burden and provide survival benefits for patients with metastatic BC (31). Studies have shown that chemotherapy or radiotherapy in FBC patients with BM could alleviate cancer-related symptoms and improve survival benefits (11, 12). However, in our multivariate analysis, chemotherapy and radiotherapy did not significantly enhance the prognosis in MBCBM patients (P>0.05). Thankfully, our conclusion was not exceptional and was supported by Zhou et al. (14). Of course, this could be owing to the absence of critical information in the SEER database, such as therapeutic drugs and doses, patient responsiveness, and compliance, preventing in-depth analysis.

During the coronavirus disease 2019 (COVID-19) pandemic, clinical predictive models *via* artificial intelligence(AI) algorithms have effectively facilitated the diagnosis and prognostic process and accurately anticipated epidemic peaks and trends (32). Such personalized, intelligent medical services might provide new insights into improving the prognosis and survival of rare diseases, especially malignant tumors (33). The therapy of MBC patients is frequently extrapolated from the treatment guidelines for postmenopausal patients with FBC due to a lack of targeted clinical trials and enough evidence-based data (26). As a result, the standardization of MBCBM treatment in male patients has not been as evident as in female patients (3). Therefore, identifying BM risk factors and predicting survival time is crucial for individualized treatment selection in MBC patients. Previous research has shown that algorithms in the X-tile software are able to determine reliable, optimal cut points for constant variables. Camp et al. applied X-tile analysis to reveal a continuous distribution of tumor size and U-shaped age distribution of age in breast cancer patients, and visualized the best cut points for prognostic markers such as growth factor receptor 2 and estrogen receptor (23). Wei et al. applied X-tile software to reveal the association between CpG methylation and survival in clear cell renal cell carcinoma patients, determine the optimal cutoff value, and divide high-risk and low-risk groups (24). We developed two risk stratification systems for OS and CSS in this study based on the above two prognostic nomograms. The KM survival curves revealed significant differences among the three risk groups, proving the validity of risk stratification systems. Following that, we established three visualized dynamic nomograms with an easy-to-use interface that allowed users to intuitively comprehend the risk of BM in MBC patients as well as the survival probability of MBCBM patients. Doctors and patients could use visualized dynamic nomograms and risk stratification systems to estimate tumor risk and adapt treatment strategies immediately.

It’s important to note that this research has several limitations: First, this is a retrospective study, and selection bias is unavoidable. Second, since 2010, when the SEER database started collecting and integrating data on distant metastases, previous instances were omitted, limiting the sample size. Third, some potentially crucial characteristics, such as the number and location of bone metastases and information on endocrine therapy and targeted therapy, are unavailable from the SEER database, affecting outcomes’ reliability. Fourth, despite the study’s nomograms’ excellent predictive performance after internal validation, more MBCBM patients’ data from medical centers worldwide is still required for external validation.

## Conclusion

We first developed and validated novel nomograms that could objectively predict the diagnosis and prognosis of MBCBM patients, with more excellent predictive performance and clinical utility than the TNM staging system. Furthermore, prognostic-based risk stratification systems and visualized dynamic nomograms could assist doctors and patients in quickly executing tailored risk assessments and clinical decision-making, as well as devising optimal treatment and follow-up plans.

## Data availability statement

Publicly available datasets were analyzed in this study. This data can be found here: https://seer.cancer.gov.

## Ethics statement

This study does not require ethical review and approval. The SEER database is an open online database for free download and analysis by researchers worldwide. All patient data is anonymized and de-identified. In accordance with national legislation and institutional requirements, this study does not require written informed consent.

## Author contributions

BG and FH designed this study. BG and M-DW collected data, analyzed statistics, and draw graphs. X-LO and M-FL drafted the manuscript. FH proofread the manuscript. All authors approved the final version of the manuscript.

## Funding

This study was supported by the Natural Science Foundation of Jilin Province (No. 20210101296JC).

## Conflict of interest

The authors declare that the research was conducted in the absence of any commercial or financial relationships that could be construed as a potential conflict of interest.

## Publisher’s note

All claims expressed in this article are solely those of the authors and do not necessarily represent those of their affiliated organizations, or those of the publisher, the editors and the reviewers. Any product that may be evaluated in this article, or claim that may be made by its manufacturer, is not guaranteed or endorsed by the publisher.

## Supplementary material

Supplementary Figure S1 A visualized dynamic diagnostic nomogram for estimating the risk of BM in MBC patients. 95% confidence interval for the probabilities of estimating the risk of BM in this patient under these conditions (T1 stage, T2 stage, T3 stage, T4 stage) **(B)**. Numerical summary for estimating the risk of BM in this patient under the above conditions **(C)**. Due to a large number of visitors to the webpage, if the application cannot be used normally, please click "Quilt" or "Reload" in the lower-left corner to try again.

Supplementary Figure S2 A visualized dynamic prognostic nomogram for predicting the OS in MBCBM patients. The curve of the predicted probability of survival for this patient over time **(A)**. 95% confidence intervals of the 12-, 24-, 36-, 48-, and 60months OS survival probabilities for this patient **(B)**. Numerical summary for prognostic analysis of this patient at different OS **(C)**.

Supplementary Figure S3 A visualized dynamic prognostic nomogram for predicting the CSS in MBCBM patients. The curve of the predicted probability of survival for this patient over time **(A)**. 95% confidence intervals of the 12-, 24-, 36-, 48-, and 60months CSS survival probabilities for this patient **(B)**. Numerical summary for prognostic analysis of this patient at different CSS **(C)**.
