# Supplementary material for: Risk stratification system and visualized dynamic nomogram constructed for predicting diagnosis and prognosis in rare male breast cancer patients with bone metastases
Source: Front Endocrinol (Lausanne). 2022 Nov 11;13:1013338. doi: 10.3389/fendo.2022.1013338 (PMC9691876; doi:10.3389/fendo.2022.1013338)
Supplement: Supplementary file 1 [file Table_1.docx]

**Supplementary Table S1**

**Simple and multivariate logistic regression analysis of risk factors for BM in MBC patients.**

| **Characteristics** | Univariate analysis | |  | Multivariate analysis | |
| --- | --- | --- | --- | --- | --- |
|  | OR (95%CI) | P |  | OR (95%CI) | P |
| **Age(years)** |  |  |  |  |  |
| ≥80 | Reference |  |  | Reference |  |
| 20-39 | 5.584(2.518-12.383) | **<0.001** |  | 5.453(2.568-11.162） | **<0.001** |
| 40-59 | 2.089(1.253-3.482) | **0.005** |  | 1.678(1.07-2.693） | 0.064 |
| 60-79 | 1.58(0.981-2.544) | 0.06 |  | 1.577(1.048-2.446） | 0.076 |
| **Race** |  |  |  |  |  |
| Black | Reference |  |  | Reference |  |
| Others | 0.889(0.473-1.672) | 0.715 |  | 1.136(0.624-1.997） | 0.718 |
| White | 0.657(0.454-0.95) | **0.026** |  | 1.235(0.879-1.764） | 0.317 |
| **Marital status** |  |  |  |  |  |
| Alone | Reference |  |  | Reference |  |
| Married | 0.513(0.383-0.688) | **<0.001** |  | 0.707(0.54-0.928） | **0.035** |
| **Year of diagnosis** |  |  |  |  |  |
| 2010-2014 | Reference |  |  |  |  |
| 2015-2019 | 1.09(0.814-1.46) | 0.562 |  |  |  |
| **Primary site** |  |  |  |  |  |
| Breast, NOS | Reference |  |  | Reference |  |
| Central portion | 0.441(0.301-0.646) | **<0.001** |  | 0.692(0.482-1.003） | 0.097 |
| Overlapping lesion | 0.461(0.282-0.754) | **0.002** |  | 0.763(0.48-1.204） | 0.331 |
| Peripheral portion | 0.462(0.295-0.726) | **0.001** |  | 0.871(0.567-1.34） | 0.597 |
| **Histological subtype** |  |  |  |  |  |
| Infiltrating duct carcinoma | Reference |  |  | Reference |  |
| Others | 0.578(0.348-0.958) | **0.034** |  | 0.623(0.386-0.962） | 0.086 |
| **Breast cancer subtype** |  |  |  |  |  |
| Luminal A | Reference |  |  | Reference |  |
| Luminal B | 1.822(1.23-2.7) | **0.003** |  | 1.242(0.858-1.767） | 0.323 |
| Others | 2.205(1.38-3.524) | **0.001** |  | 1.452(0.795-2.506） | 0.283 |
| **ER status** |  |  |  |  |  |
| Negative | Reference |  |  | Reference |  |
| Positive | 0.288(0.161-0.516) | **<0.001** |  | 0.493(0.228-1.073） | 0.132 |
| **PR status** |  |  |  |  |  |
| Negative | Reference |  |  | Reference |  |
| Positive | 0.451(0.305-0.667) | **<0.001** |  | 0.689(0.458-1.063） | 0.144 |
| **Laterality** |  |  |  |  |  |
| Left | Reference |  |  |  |  |
| Right | 1.073(0.803-1.435) | 0.632 |  |  |  |
| **Tumor size(mm)** |  |  |  |  |  |
| <20 | Reference |  |  | Reference |  |
| >50 | 29.229(16.997-50.263) | **<0.001** |  | 6.047(2.833-12.947） | **<0.001** |
| 20-50 | 4.544(2.747-7.515) | **<0.001** |  | 2.21(1.122-4.306） | 0.052 |
| **Grade** |  |  |  |  |  |
| Grade I | Reference |  |  | Reference |  |
| Grade II | 2.402(1.204-4.794) | **0.013** |  | 1.528(0.859-2.949） | 0.255 |
| Grade III/IV | 3.462(1.73-6.927) | **<0.001** |  | 1.5(0.834-2.917） | 0.283 |
| **T stage** |  |  |  |  |  |
| T1 | Reference |  |  | Reference |  |
| T2 | 3.539(2.189-5.721) | **<0.001** |  | 1.441(0.785-2.817） | 0.347 |
| T3 | 20.909(11.528-37.926) | **<0.001** |  | 2.756(1.25-6.238） | **0.038** |
| T4 | 19.021(11.583-31.236) | **<0.001** |  | 4.32(2.291-8.459） | **<0.001** |
| **N stage** |  |  |  |  |  |
| N0 | Reference |  |  | Reference |  |
| N1 | 4.363(3.013-6.319) | **<0.001** |  | 2.767(1.987-3.892） | **<0.001** |
| N2 | 5.434(3.382-8.732) | **<0.001** |  | 2.609(1.68-4.026） | **<0.001** |
| N3 | 7.74(4.545-13.183) | **<0.001** |  | 3.232(1.949-5.276） | **<0.001** |

**Bold values refer to P < 0.05 with statistical significance**
