# Supplementary material for: Risk stratification system and visualized dynamic nomogram constructed for predicting diagnosis and prognosis in rare male breast cancer patients with bone metastases
Source: Front Endocrinol (Lausanne). 2022 Nov 11;13:1013338. doi: 10.3389/fendo.2022.1013338 (PMC9691876; doi:10.3389/fendo.2022.1013338)
Supplement: Supplementary file 2 [file Table_2.docx]

**Supplementary Table S2**

**Simple COX regression analysis for OS and CSS in MBCBM patients.**

| **Characteristics** | OS | |  | CSS | |
| --- | --- | --- | --- | --- | --- |
|  | HR (95%CI) | P |  | HR (95%CI) | P |
| **Age(years)** |  |  |  |  |  |
| ≥80 | Reference |  |  | Reference |  |
| 20-39 | 0.253（0.088-0.73） | **0.033** |  | 0.215（0.059-0.783） | **0.05** |
| 40-59 | 0.711（0.416-1.216） | 0.296 |  | 0.825（0.444-1.531） | 0.608 |
| 60-79 | 0.61（0.367-1.012） | 0.108 |  | 0.715（0.393-1.301） | 0.357 |
| **Race** |  |  |  |  |  |
| Black | Reference |  |  | Reference |  |
| Others | 1.74（0.875-3.459） | 0.185 |  | 1.719（0.82-3.603） | 0.228 |
| White | 1.111（0.75-1.647） | 0.659 |  | 1.053（0.684-1.622） | 0.844 |
| **Marital status** |  |  |  |  |  |
| Alone | Reference |  |  | Reference |  |
| Married | 1.081（0.796-1.468） | 0.676 |  | 1.165（0.831-1.633） | 0.456 |
| **Year of diagnosis** |  |  |  |  |  |
| 2010-2014 | Reference |  |  | Reference |  |
| 2015-2019 | 0.945（0.677-1.318） | 0.779 |  | 0.901（0.629-1.29） | 0.633 |
| **Primary site** |  |  |  |  |  |
| Breast, NOS | Reference |  |  | Reference |  |
| Central portion | 0.65（0.446-0.946） | 0.059 |  | 0.612（0.405-0.925） | **0.05** |
| Overlapping lesion | 0.919（0.552-1.53） | 0.786 |  | 0.978（0.582-1.643） | 0.943 |
| Peripheral portion | 0.75（0.473-1.189） | 0.305 |  | 0.792（0.494-1.271） | 0.417 |
| **Histological subtype** |  |  |  |  |  |
| Infiltrating duct carcinoma | Reference |  |  | Reference |  |
| Others | 0.891（0.542-1.464） | 0.701 |  | 0.882（0.525-1.483） | 0.692 |
| **Breast cancer subtype** |  |  |  |  |  |
| Luminal A | Reference |  |  | Reference |  |
| Luminal B | 1.001（0.675-1.485） | 0.995 |  | 1.063（0.691-1.636） | 0.815 |
| Others | 2.691（1.756-4.124） | **<0.001** |  | 3.137（2.009-4.897） | **<0.001** |
| **ER status** |  |  |  |  |  |
| Negative | Reference |  |  | Reference |  |
| Positive | 0.071（0.04-0.126） | **<0.001** |  | 0.069（0.038-0.124） | **<0.001** |
| **PR status** |  |  |  |  |  |
| Negative | Reference |  |  | Reference |  |
| Positive | 0.282（0.191-0.415） | **<0.001** |  | 0.276（0.183-0.416） | **<0.001** |
| **Laterality** |  |  |  |  |  |
| Left | Reference |  |  | Reference |  |
| Right | 1.198（0.886-1.621） | 0.325 |  | 1.182（0.854-1.637） | 0.398 |
| **Tumor size(mm)** |  |  |  |  |  |
| <20 | Reference |  |  | Reference |  |
| >50 | 1.639（0.893-3.008） | 0.181 |  | 1.439（0.775-2.67） | 0.333 |
| 20-50 | 1.499（0.836-2.69） | 0.254 |  | 1.462（0.81-2.637） | 0.29 |
| **Grade** |  |  |  |  |  |
| Grade I | Reference |  |  | Reference |  |
| Grade II | 1.01（0.429-2.379） | 0.985 |  | 0.915（0.385-2.174） | 0.865 |
| Grade III/IV | 1.098（0.469-2.572） | 0.856 |  | 1.108（0.472-2.601） | 0.843 |
| **T stage** |  |  |  |  |  |
| T1 | Reference |  |  | Reference |  |
| T2 | 1.532（0.899-2.611） | 0.188 |  | 1.486（0.833-2.651） | 0.261 |
| T3 | 2.543（1.382-4.68） | **0.012** |  | 2.3（1.187-4.455） | **0.038** |
| T4 | 1.24（0.721-2.133） | 0.513 |  | 1.038（0.57-1.887） | 0.919 |
| **N stage** |  |  |  |  |  |
| N0 | Reference |  |  | Reference |  |
| N1 | 1.027（0.698-1.511） | 0.911 |  | 1.036（0.676-1.586） | 0.892 |
| N2 | 0.863（0.528-1.411） | 0.621 |  | 0.796（0.463-1.367） | 0.487 |
| N3 | 0.599（0.342-1.051） | 0.134 |  | 0.535（0.289-0.991） | 0.095 |
| **Brain metastasis** |  |  |  |  |  |
| No | Reference |  |  | Reference |  |
| Yes | 1.755（0.92-3.348） | 0.152 |  | 1.706（0.849-3.427） | 0.208 |
| **Lung metastasis** |  |  |  |  |  |
| No | Reference |  |  | Reference |  |
| Yes | 0.865（0.624-1.199） | 0.465 |  | 0.823（0.575-1.179） | 0.373 |
| **Liver metastasis** |  |  |  |  |  |
| No | Reference |  |  | Reference |  |
| Yes | 1.315（0.808-2.142） | 0.355 |  | 1.392（0.851-2.277） | 0.269 |
| **Surgery** |  |  |  |  |  |
| No | Reference |  |  | Reference |  |
| Breast-conserving surgery | 0.825（0.42-1.622） | 0.639 |  | 0.885（0.448-1.75） | 0.768 |
| Partial mastectomy | 0.698（0.446-1.092） | 0.186 |  | 0.826（0.499-1.365） | 0.531 |
| Radical mastectomy | 0.607（0.409-0.902） | **0.038** |  | 0.586（0.38-0.903） | **0.042** |
| **Radiation** |  |  |  |  |  |
| No | Reference |  |  | Reference |  |
| Yes | 0.968（0.713-1.316） | 0.863 |  | 0.947（0.678-1.322） | 0.787 |
| **Chemotherapy** |  |  |  |  |  |
| No | Reference |  |  | Reference |  |
| Yes | 0.874（0.647-1.181） | 0.462 |  | 0.911（0.657-1.261） | 0.636 |

**Bold values refer to P≤0.05 with statistical significance**
