# Supplementary material for: Risk stratification system and visualized dynamic nomogram constructed for predicting diagnosis and prognosis in rare male breast cancer patients with bone metastases
Source: Front Endocrinol (Lausanne). 2022 Nov 11;13:1013338. doi: 10.3389/fendo.2022.1013338 (PMC9691876; doi:10.3389/fendo.2022.1013338)
Supplement: Supplementary file 3 [file Table_3.docx]

**Supplementary Table S3**

**Multivariate COX regression analysis for OS and CSS in MBCBM patients.**

| **Characteristics** | OS | |  | CSS | |
| --- | --- | --- | --- | --- | --- |
|  | HR (95%CI) | P |  | HR (95%CI) | P |
| **Age(years)** |  |  |  |  |  |
| ≥80 | Reference |  |  | Reference |  |
| 20-39 | 0.2 (0.0533-0.748) | **0.017** |  | 0.160 (0.032-0.797) | **0.025** |
| 40-59 | 0.632 (0.319-1.251) | 0.188 |  | 0.693 (0.314-1.531) | 0.365 |
| 60-79 | 0.425 (0.225-0.803) | **0.008** |  | 0.446 (0.212-0.941) | **0.034** |
| **Primary site** |  |  |  |  |  |
| Breast, NOS |  |  |  | Reference |  |
| Central portion |  |  |  | 0.649 (0.366-1.151) | 0.139 |
| Overlapping lesion |  |  |  | 0.763 (0.391-1.486) | 0.426 |
| Peripheral portion |  |  |  | 0.829 (0.423-1.625) | 0.585 |
| **Breast cancer subtype** |  |  |  |  |  |
| Luminal A | Reference |  |  | Reference |  |
| Luminal B | 1.009 (0.605-1.683) | 0.973 |  | 0.999 (0.562-1.773) | 0.996 |
| Others | 0.998 (0.487-2.044) | 0.995 |  | 1.119 (0.508-2.467) | 0.780 |
| **ER status** |  |  |  |  |  |
| Negative | Reference |  |  | Reference |  |
| Positive | 0.071 (0.027-0.189) | **<0.001** |  | 0.078 (0.027-0.223) | **<0.001** |
| **PR status** |  |  |  |  |  |
| Negative | Reference |  |  | Reference |  |
| Positive | 0.499 (0.278-0.896) | **0.020** |  | 0.506 (0.265-0.964) | **0.038** |
| **T stage** |  |  |  |  |  |
| T1 | Reference |  |  | Reference |  |
| T2 | 1.946 (0.998-3.793) | 0.051 |  | 2.094 (1.003-4.372) | **0.049** |
| T3 | 3.449 (1.616-7.36) | **0.001** |  | 3.379 (1.448-7.887) | **0.005** |
| T4 | 1.61 (0.81-3.202) | 0.174 |  | 1.356 (0.623-2.95) | 0.443 |
| **Surgery** |  |  |  |  |  |
| No | Reference |  |  | Reference |  |
| Breast-conserving surgery | 0.567 (0.248-1.299) | 0.180 |  | 0.509 (0.211-1.224) | 0.132 |
| Partial mastectomy | 0.689 (0.394-1.206) | 0.192 |  | 0.693 (0.356-1.348) | 0.280 |
| Radical mastectomy | 0.445 (0.27-0.735) | **0.002** |  | 0.402 (0.231-0.701) | **0.001** |

**Bold values refer to P < 0.05 with statistical significance**
