# Supplementary figures and images for: Risk stratification system and visualized dynamic nomogram constructed for predicting diagnosis and prognosis in rare male breast cancer patients with bone metastases
Source: Front Endocrinol (Lausanne). 2022 Nov 11;13:1013338. doi: 10.3389/fendo.2022.1013338 (PMC9691876; doi:10.3389/fendo.2022.1013338)

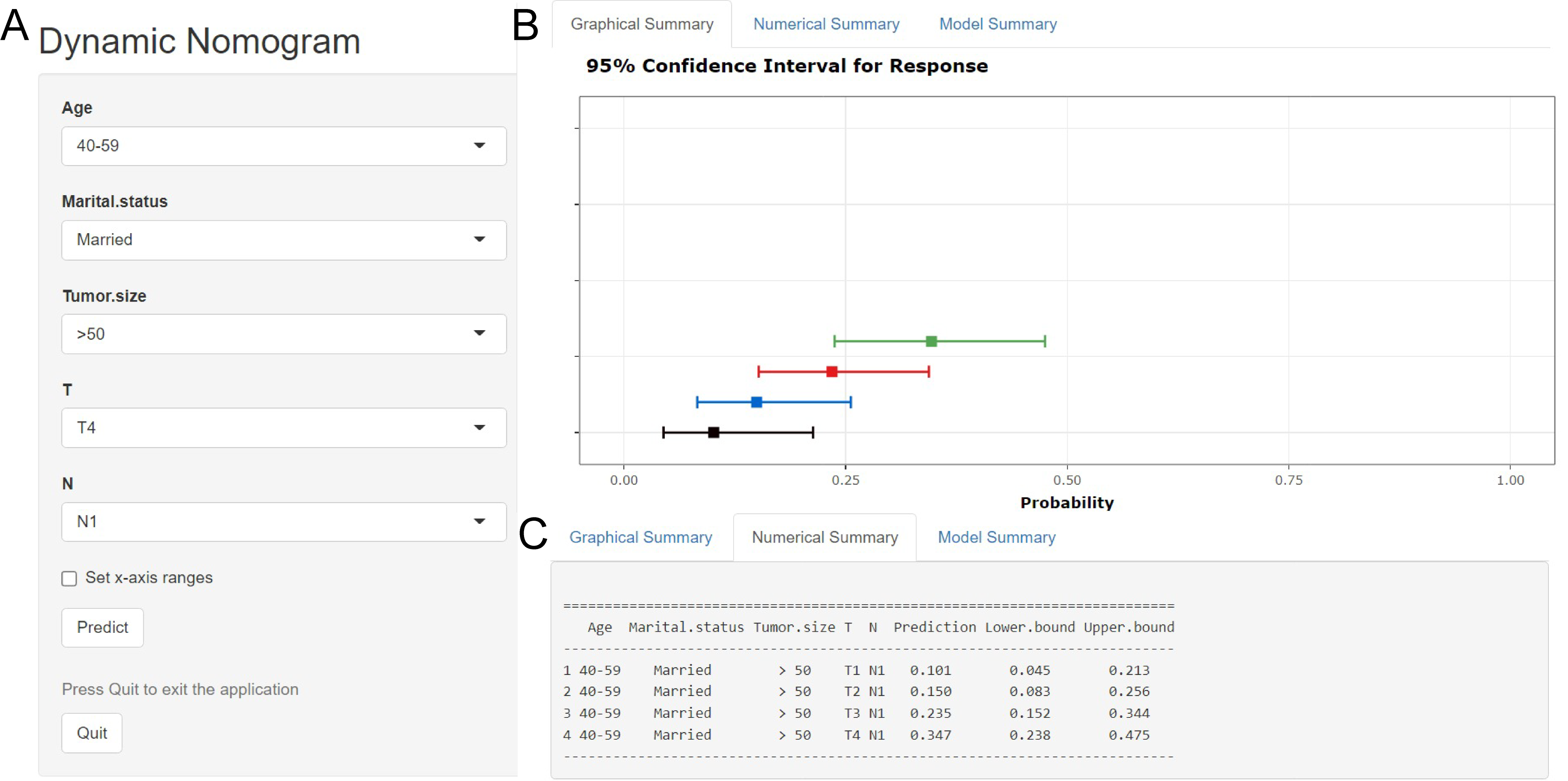

Supplement: Supplementary Figure S1 — A visualized dynamic diagnostic nomogram for estimating the risk of BM in MBC patients. 95% confidence interval for the probabilities of estimating the risk of BM in this patient under these conditions (T1 stage, T2 stage, T3 stage, T4 stage) (B). Numerical summary for estimating the risk of BM in this patient under the above conditions (C). Due to a large number of visitors to the webpage, if the application cannot be used normally, please click "Quilt" or "Reload" in the lower-left corner to try again. [file Image_1.tif]

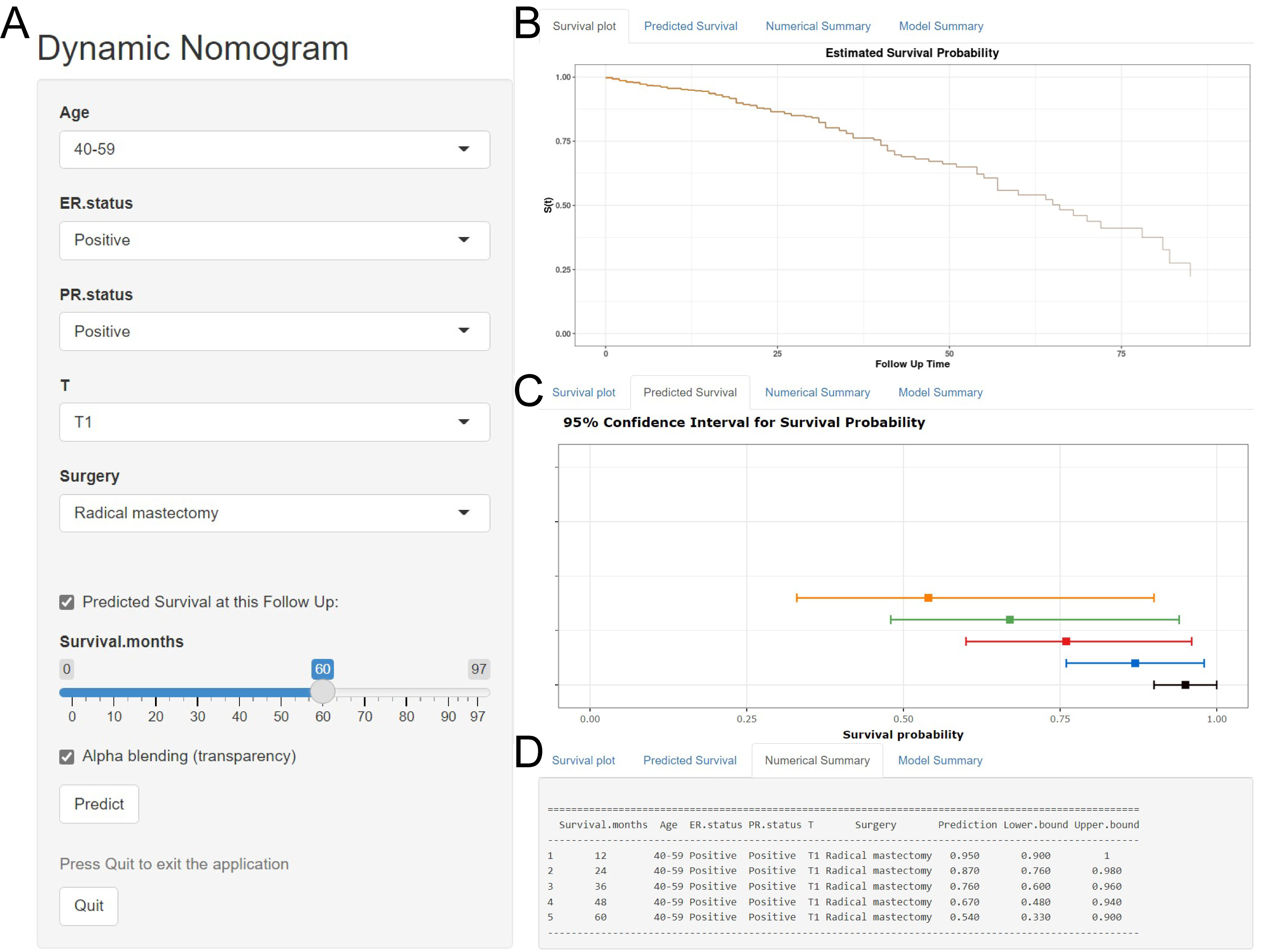

Supplement: Supplementary Figure S2 — A visualized dynamic prognostic nomogram for predicting the OS in MBCBM patients. The curve of the predicted probability of survival for this patient over time (A). 95% confidence intervals of the 12-, 24-, 36-, 48-, and 60months OS survival probabilities for this patient (B). Numerical summary for prognostic analysis of this patient at different OS (C). [file Image_2.tif]

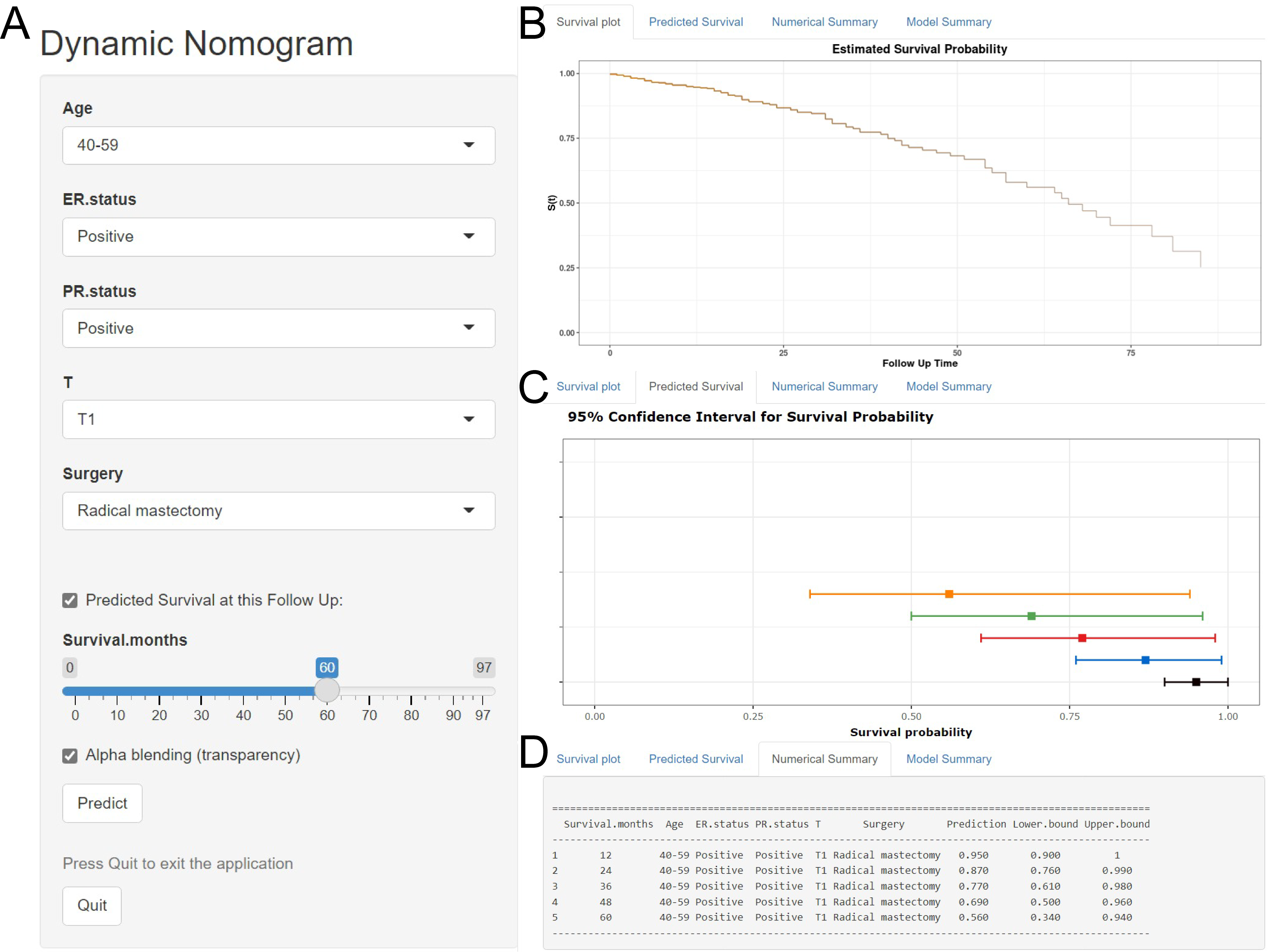

Supplement: Supplementary Figure S3 — A visualized dynamic prognostic nomogram for predicting the CSS in MBCBM patients. The curve of the predicted probability of survival for this patient over time (A). 95% confidence intervals of the 12-, 24-, 36-, 48-, and 60months CSS survival probabilities for this patient (B). Numerical summary for prognostic analysis of this patient at different CSS (C). [file Image_3.tif]
